# Presymptomatic grey matter alterations in ALS kindreds: a computational neuroimaging study of asymptomatic *C9orf72* and *SOD1* mutation carriers

**DOI:** 10.1007/s00415-023-11764-5

**Published:** 2023-05-13

**Authors:** Peter Bede, Dorothée Lulé, Hans-Peter Müller, Ee Ling Tan, Johannes Dorst, Albert C. Ludolph, Jan Kassubek

**Affiliations:** 1 Computational Neuroimaging Group (CNG), School of Medicine, Trinity College Dublin, Dublin, D02 RS90 Ireland; 2 Department of Neurology, St James’s Hospital, Dublin, Ireland; 3 Department of Neurology, University of Ulm, Ulm, Germany; 4 German Centre of Neurodegenerative Diseases (DZNE), Ulm, Germany

**Keywords:** Presymptomatic, Asymptomatic, Amyotrophic lateral sclerosis, Frontotemporal dementia, Magnetic resonance imaging (MRI), Biomarker, *C9orf72*, *SOD1*, Pharmaceutical trials

## Abstract

**Background:**

The characterisation of presymptomatic disease-burden patterns in asymptomatic mutation carriers has a dual academic and clinical relevance. The understanding of disease propagation mechanisms is of considerable conceptual interests, and defining the optimal time of pharmacological intervention is essential for improved clinical trial outcomes.

**Methods:**

In a prospective, multimodal neuroimaging study, 22 asymptomatic *C9orf72* GGGGCC hexanucleotide repeat carriers, 13 asymptomatic subjects with *SOD1*, and 54 “gene-negative” ALS kindreds were enrolled. Cortical and subcortical grey matter alterations were systematically appraised using volumetric, morphometric, vertex, and cortical thickness analyses. Using a Bayesian approach, the thalamus and amygdala were further parcellated into specific nuclei and the hippocampus was segmented into anatomically defined subfields.

**Results:**

Asymptomatic GGGGCC hexanucleotide repeat carriers in *C9orf72* exhibited early subcortical changes with the preferential involvement of the pulvinar and mediodorsal regions of the thalamus, as well as the lateral aspect of the hippocampus. Volumetric approaches, morphometric methods, and vertex analyses were anatomically consistent in capturing focal subcortical changes in asymptomatic *C9orf72* hexanucleotide repeat expansion carriers. *SOD1* mutation carriers did not exhibit significant subcortical grey matter alterations. In our study, none of the two asymptomatic cohorts exhibited cortical grey matter alterations on either cortical thickness or morphometric analyses.

**Discussion:**

The presymptomatic radiological signature of *C9orf72* is associated with selective thalamic and focal hippocampal degeneration which may be readily detectable before cortical grey matter changes ensue. Our findings confirm selective subcortical grey matter involvement early in the course of *C9orf72*-associated neurodegeneration.

**Supplementary Information:**

The online version contains supplementary material available at 10.1007/s00415-023-11764-5.

## Introduction

One of the important paradigm shifts in amyotrophic lateral sclerosis (ALS) research is the departure from the concept of “one-drug for all” to the pursuit of precision, genotype-specific pharmacological interventions [1, 2]. The recognition of the fundamental heterogeneity of ALS led to the nuanced characterisation of various ALS genotypes and phenotypes [3–6]. It is increasingly recognised that symptom manifestation in ALS is preceded by a long presymptomatic phase [7] and degenerative changes may be detected decades before symptom manifestation [8, 9]. The ideal timing of therapeutic intervention should therefore be reconsidered, especially in genetically susceptible cohorts. Antisense oligonucleotide (ASO)-therapies have been approved for the treatment of spinal muscular atrophy and Duchenne muscular dystrophy [10–12], and also trialled in ALS [1]. Accordingly, the assessment of disease burden prior to symptom manifestation is of pressing practical relevance. Two most commonly studied genotypes in ALS are the GGGGCC hexanucleotide repeat expansions (HRE) in *C9orf72* and *SOD1*. *C9orf72* HRE may lead to a spectrum of clinical manifestations spanning from ALS to frontotemporal dementia (FTD) and clinical manifestations are thought to be closely associated with patterns of phosphorylated 43 kDa TAR DNA-binding protein (pTDP-43) burden. Striatal [13], temporal [14], frontal [15], cerebellar [16], and thalamic [13, 16] grey matter alterations have previously been described in asymptomatic *C9orf72* cohorts. Presymptomatic orbitofrontal [17], corpus callosum, cingulate, uncinate [8, 13, 18], and corticospinal tract [9, 13] white matter changes have also been consistently detected and PET studies captured frontotemporal, thalamic, and basal ganglia hypometabolism [19]. Three mechanisms have been proposed for *C9orf72* HRE-associated pathophysiology; loss of *C9orf72* function through haploinsufficiency, toxic gain-of-function due to the generation of aberrant HRE-containing RNA, and toxic gain-of-function through the accumulation of dipeptide repeat proteins translated from hexanucleotide repeat RNA [20]. These mechanisms are thought to trigger a multitude of cellular responses, of which pTDP-43 accumulation may only be one amongst several processes. Accordingly, cerebral involvement outside the neocortex may represent a distinguishing feature of *C9orf72* distinct from other genetic variants, such as *SOD1* mutations [21]. The presymptomatic imaging signature of *SOD1* is thought to be relatively unique; white matter changes in the posterior limb of the internal capsule [22], reduced superior spinal cord NAA/Cr and NAA/Myo ratios [23], and reduced left frontotemporal junction flumazenil binding [24], and have been reported. While structural, metabolic, and function thalamic [8, 13, 16, 18, 25–30] changes have been previously described in presymptomatic *C9orf72* hexanucleotide carriers and longitudinal thalamic changes have also been explored [25, 31, 32], the predilection for specific thalamic nuclei remains poorly characterised despite the unique role of thalamic nuclei in relaying specific sensory, cognitive, and behavioural functions [33–36]. Presymptomatic amygdalar and hippocampal alterations are also under evaluated despite the selective involvement of these structures in symptomatic mutation carriers [37–41]. Accordingly, the principal objective of this study is the nuanced characterisation of cortical and subcortical grey matter changes in two cohorts of presymptomatic mutation carriers using a panel of supplementary imaging techniques. Our hypothesis is that presymptomatic hexanucleotide expansion carriers exhibit focal subcortical degeneration with concomitant alterations in their cortical projection areas.

## Methods

The study was approved by the Ethics Committee of the University of Ulm (reference 68/19), in accordance with the ethical standards of the current version of the revised Helsinki declaration. All participants gave informed consent prior to enrolment. Recruitment strategy and genetic testing have been previously described [17].

### Neuroimaging

T1-weighted data were acquired on a 1.5 Tesla Magnetom Symphony (Siemens Medical) with a 12-channel head coil. Acquisition parameters have been described previously [42]. T2-weighted and fluid-attenuated inversion recovery (FLAIR) images were systematically reviewed for confounding vascular or neuroinflammatory pathologies. Raw T1-weighted MR data were screened for artifacts, developmental malformations, arachnoid or porencephalic cysts, hydrocephalus, or other pathologies that could impact on quantitative morphometric analyses prior to pre-processing.

### Volumetric analyses

The standard pre-processing steps of the FreeSurfer image analysis suite [43] were first implemented, including removal of non-brain tissue, segmentation of the subcortical white matter and deep grey matter structures, intensity normalization, tessellation of the grey matter–white matter boundary, and automated topology correction. Following quality control steps for segmentation accuracy, overall volumes of subcortical structures and total intracranial volume estimates (eTIV) were retrieved from each subject. Total volume estimates of the following structures were generated in the left and right hemispheres separately: thalamus, caudate, putamen, pallidum, hippocampus, amygdala, and nucleus accumbens.

### Nuclear segmentation of the thalamus and amygdala

The thalamus was segmented into 25 subregions using Bayesian inference based on a probabilistic atlas developed upon histological data [44]. The thalamus was first parcellated into the following nuclei in each hemisphere: anteroventral (AV), laterodorsal (LD), lateral posterior (LP), ventral anterior (VA), ventral anterior magnocellular (VA mc), ventral lateral anterior (VLa), ventral lateral posterior (VLp), ventral posterolateral (VPL), ventromedial (VM), central medial (CeM), central lateral (CL), paracentral (Pc), centromedian (CM), parafascicular (Pf), paratenial (Pt), reuniens/medial ventral (MV-re), mediodorsal medial magnocellular (MDm), mediodorsal lateral parvocellular (MDl), lateral geniculate (LGN), medial geniculate (MGN), limitans/suprageniculate (L-SG), pulvinar anterior (PuA), pulvinar medial (PuM), pulvinar lateral (PuL), and pulvinar inferior (PuI). The raw volume estimates of the above nuclei were averaged between left and right and merged into the following 10 core group of nuclei defined based on their distinctive physiological function: “anteroventral”, “lateral geniculate”, “medial geniculate”, “pulvinar-limitans” (PuA, PuM, PuL, PuI, L-SG), “laterodorsal”, “lateroposterior”, “mediodorsal-paratenial-reuniens” (MDm, MDl, MV-re, Pt), “motor nuclei” (VA, VAmc, VLa, VLp), “sensory nuclei” (VPL, VM), and “intralaminar” (CeM, CL, Pc, CM, Pf). “Total thalamic volume” was defined as the mean of the left and right thalamus volume estimates and used as a covariate in the relevant statistical models. Post hoc statistics were corrected for demographic variables, total thalamic volume, and multiple testing.

A Bayesian inference was also used to parcellate the amygdala into nine subregions using a probabilistic atlas developed based on histological data [44, 45]. The amygdala was segmented into the following nuclei in each hemisphere: lateral nucleus (LN), basal nucleus (BN), accessory basal nucleus (ABN), anterior amygdaloid area (AAA), central nucleus (CN), medial nucleus (MN), cortical nucleus (CN), cortico-amygdaloid transition (CAT), and paralaminar nucleus (PN). The raw volume estimates of the above nuclei were averaged between left and right. “Total amygdala volume” was defined as the mean of the left and right total amygdala volume estimates. Post hoc statistics were corrected for demographic variables, total amygdala volume, and multiple testing.

### Hippocampal subfield parcellation

**Fig. 1:**
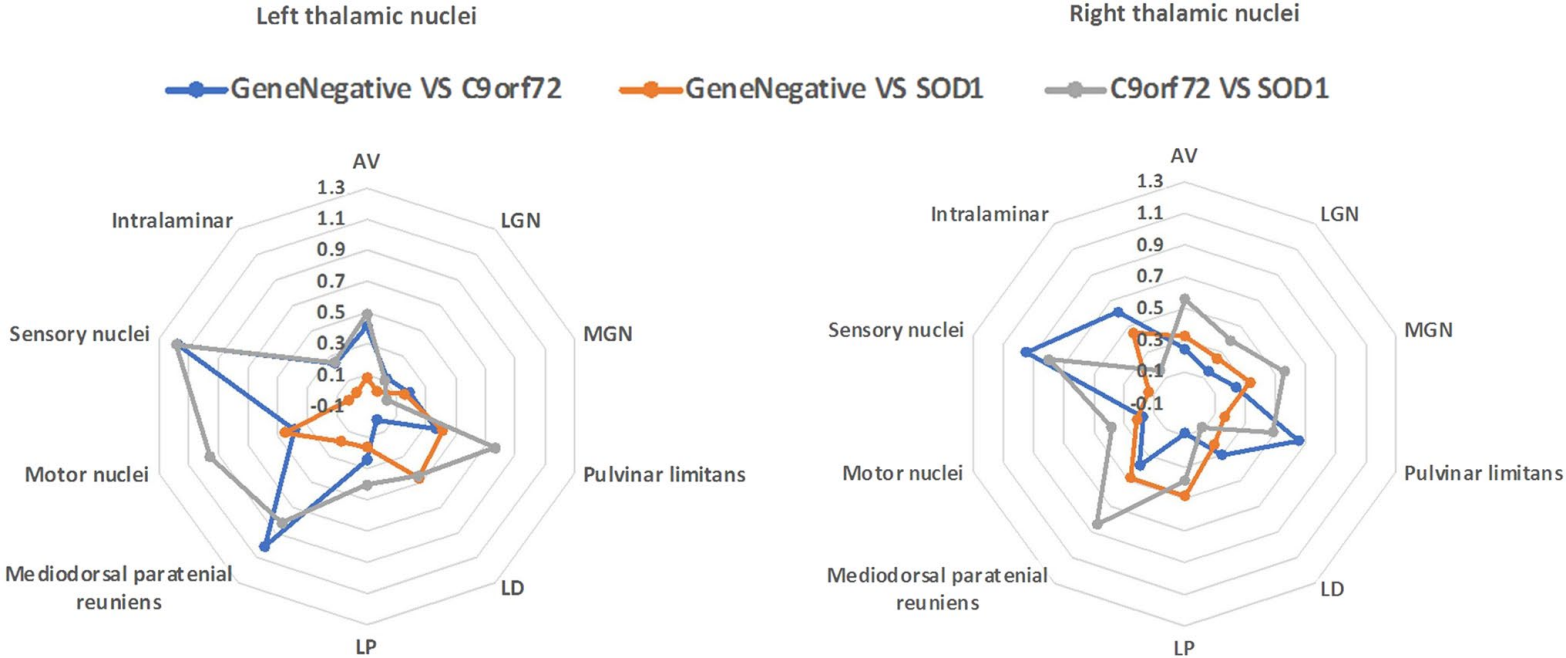
Effect-size differences in thalamic nuclei volumes between *C9orf72* hexanucleotide repeat expansion carriers, gene-negative controls and *SOD1* mutation carriers in the two thalami. *AV* anteroventral nuclei, *C9orf72* chromosome 9 open-reading frame 72, *LD* laterodorsal nuclei, *LGN* lateral geniculate, *LP* lateral posterior nuclei, *MGN* medial geniculate nuclei

The hippocampus was segmented into cytologically-defined subfields (Fig. 1A) using the FreeSurfer image analysis suite [43]. The pre-processing pipeline included the removal of non-brain tissue, segmentation of the subcortical white matter and deep grey matter structures, intensity normalization, tessellation of the grey matter–white matter boundary, and automated topology correction. The hippocampal stream of the FreeSurfer package was used for the delineation of the following hippocampal subfields: CA1, CA2/3, CA4, fimbria, hippocampal fissure, presubiculum, subiculum, hippocampal tail, parasubiculum, molecular layer; granule cell layer of the dentate gyrus (GC-DG), and hippocampal–amygdala transition area (HATA) [46].

### Vertex analyses

Surface projected patterns of atrophy were evaluated using vertex analyses. As described previously [47], FMRIB’s subcortical segmentation and registration tool FIRST [48] was utilised to characterise focal thalamic shape deformations. Vertex locations of each participant were projected on the surface of an average thalamic shape template as scalar values, positive value being outside the surface, and negative values inside. Using study-specific design matrices specifying group membership and covariates, permutation-based non-parametric inference was implemented for group comparisons using FMRIB’s ‘RANDOMISE’ module [49]. The design matrices included demeaned age, sex, education, and total intracranial volumes as covariates [49].

### Subcortical morphometry

FMRIB’s software library was used for brain extraction and tissue-type segmentation. Resulting grey-matter partial volume images were then aligned to MNI152 standard space using affine registration. A study-specific template was subsequently created, to which the grey matter images from each subject were non-linearly coregistered. Group membership and covariates were specified in study-specific design matrices and demeaned covariates included age, sex, education, and TIV. A voxelwise generalized linear model and permutation-based non-parametric testing was used to highlight density alterations in a merged subcortical grey matter mask accounting for multiple testing, age, sex, and education. [49, 50] Labels of the Harvard–Oxford subcortical probabilistic structural atlas was used to generate a merged subcortical grey mask incorporating the left and right caudate, thalamus, accumbens, hippocampus, amygdala, putamen, and pallidum. [51, 52]

### Cortical grey matter analyses

A dual pipeline was implemented exploring (1) cortical thickness alterations and (2) morphometric changes using voxel-based morphometry (VBM). Following pre-processing and cortical segmentation in FreeSurfer, average cortical thickness values have been retrieved from 34 cortical regions in each hemisphere separately as per the Desikan–Killiany atlas. Cortical thickness values in the following lobes and corresponding subregions regions were appraised, Frontal lobe (13 ROIs): Superior Frontal, Rostral and Caudal Middle Frontal, Pars Opercularis, Pars Triangularis, and Pars Orbitalis, Lateral and Medial Orbitofrontal, Precentral, Paracentral, Frontal Pole, Rostral Anterior cingulate, Caudal Anterior cingulate, Parietal lobe (7 ROIs): Superior Parietal, Inferior Parietal, Supramarginal, Postcentral, Precuneus, Posterior cingulate, cingulate isthmus, Temporal lobe (9 ROIs): Superior, Middle, and Inferior Temporal, Banks of the Superior Temporal Sulcus, Fusiform, Transverse Temporal, Entorhinal, Temporal Pole, Parahippocampal, Occipital lobe (4 ROIs): lateral Occipital, Lingual, Cuneus, Pericalcarine, and the Insula (1 ROI). In addition to cortical thickness analyses, voxel-based morphometry was also performed to evaluate anatomical patterns of signal intensity reductions in mutation carriers. Cortical grey matter morphometry analyses were conducted using FSL-VBM [53, 54]. Following brain extraction, motion-correction, and tissue-type segmentation, the resulting grey-matter partial volume images were aligned to MNI152 standard space using affine registration. A study-specific template was generated to which the grey matter images from each subject were non-linearly coregistered. Permutation-based non-parametric inference and the threshold-free cluster enhancement (TFCE) approach were utilised to test for differences between study groups controlling for age, sex, TIV, and education.

## Results

The main study groups were matched for age and education and differed in sex ratios (Table 1). We note that all statistical models were corrected for age, sex, and education.

**Table 1:** The demographic profile of study groups

	Gene negative (*n* = 54)	C9orf72 (*n* = 22)	SOD1 (*n* = 13)	Statistics (ANOVA, *χ*^2^; *p* value)
Age (yrs)	41.61 ± 11.75	45.00 ± 11.62	48.31 ± 14.45	*F* (2,86) = 1.842; *p* = 0.165
Gender (M / F)	24/30	5/17	9/4	*χ*^2^ (2) = 7.394; *p* = 0.025
Education (yrs)	14.89 ± 3.37	14.59 ± 3.02	13.23 ± 2.68	*F* (2,86) = 1.407; *p* = 0.251

While differences in overall subcortical volume differences did not reach significance (Table 2), mediodorsal-paratenial-reuniens in the left thalamus and pulvinar-limitans atrophy in the right thalamus was detected in asymptomatic *C9orf72* hexanucleotide repeat expansion carriers compared to gene-negative family members (Table 3.). Furthermore, higher sensory nuclei volumes were identified in *C9orf72* hexanucleotide repeat expansion carriers compared to both gene-negative controls and *SOD1* mutation carriers in both thalami. Effect sizes are illustrated in Fig. 1. Differences in amygdalar nuclei and hippocampal subfield volumes did not reach significance. Relevant output statistics, univariate p value, and effect sizes are summarised in supplementary tables 1–2.

**Table 2:** The volumetric profile (mm^3^) of evaluated structures [estimated marginal means ± standard error] (covariates: age, gender, education, and TIV)

	“Gene Negative”	*C9orf72*	*SOD1*	Univariate *p* value and effect size
Left				
Thalamus	8028.79 ± 76.15	7592.38 ± 121.20	8188.57 ± 160.65	*p* = 0.004; *η*^2^*p* = 0.125
Caudate	3438.21 ± 39.25	3365.10 ± 62.48	3312.67 ± 82.82	*p* = 0.325; *η*^2^*p* = 0.027
Putamen	4520.49 ± 60.39	4573.78 ± 96.12	4610.60 ± 127.40	*p* = 0.779; *η*^2^*p* = 0.006
Pallidum	1880.10 ± 24.82	1944.05 ± 39.50	1957.33 ± 52.36	*p* = 0.244; *η*^2^*p* = 0.034
Hippocampus	3985.09 ± 57.33	3833.27 ± 91.26	4073.76 ± 120.96	*p* = 0.236; *η*^2^*p* = 0.035
Amygdala	1489.68 ± 25.98	1462.62 ± 41.36	1592.96 ± 54.82	*p* = 0.160; *η*^2^*p* = 0.044
Accumbens	457.18 ± 9.66	433.20 ± 15.38	472.04 ± 20.38	*p* = 0.271; *η*^2^*p* = 0.031
Right				
Thalamus	7864.32 ± 72.02	7512.04 ± 114.63	8105.19 ± 151.93	*p* = 0.006; *η*^2^*p* = 0.117
Caudate	3476.46 ± 39.28	3460.89 ± 62.52	3388.91 ± 82.86	*p* = 0.644; *η*^2^*p* = 0.011
Putamen	4579.26 ± 57.41	4555.90 ± 91.38	4727.42 ± 121.12	*p* = 0.495; *η*^2^*p* = 0.017
Pallidum	1865.01 ± 24.24	1922.27 ± 38.58	1924.18 ± 51.14	*p* = 0.354; *η*^2^*p* = 0.025
Hippocampus	4090.66 ± 56.34	3986.73 ± 89.68	4250.73 ± 118.87	*p* = 0.220; *η*^2^*p* = 0.036
Amygdala	1664.20 ± 23.58	1645.97 ± 37.53	1726.34 ± 49.75	*p* = 0.430; *η*^2^*p* = 0.020
Accumbens	484.14 ± 10.67	470.90 ± 16.98	480.98 ± 22.51	*p* = 0.810; *η*^2^*p* = 0.005

Age = 43.43; Sex = 1.57; Education = 14.57; Total Intracranial Volume = 1585cm^3^. Post hoc comparisons were not performed, because the multivariate omnibus test was not significant: Wilks’ Lambda = 0.634; *F* (28, 138) = 1.260; *p* = 0.192. Partial *η*^2^ effect size is interpreted as small (*η*^2^*p* = 0.01), medium (*η*^2^*p* = 0.06), and large (*η*^2^*p* = 0.14)

**Table 3:** Thalamic nuclei volumes mm^3^ [estimated marginal means ± standard error] (covariates: age, sex, education, and thalamic volume)

	“Gene Negative”	*C9orf72*	*SOD1*	Univariate *p* value and effect size	Significant post hoc contrasts (Bonferroni)
Left thalamus^a^					
AV	137.06 ± 2.00	130.87 ± 3.25	138.34 ± 4.20	*p* = 0.240; *η*^2^*p* = 0.034	
LGN	166.12 ± 3.36	163.11 ± 5.47	165.70 ± 7.06	*p* = 0.899; * η*^2^*p* = 0.003	
MGN	119.64 ± 1.89	122.31 ± 3.08	121.84 ± 3.97	*p* = 0.724; * η*^2^*p* = 0.008	
Pulvinar limitans	1459.01 ± 12.86	1423.92 ± 20.91	1498.39 ± 26.98	*p* = 0.105; * η*^2^*p* = 0.054	
LD	33.19 ± 0.89	33.24 ± 1.44	36.30 ± 1.86	*p* = 0.315; * η*^2^*p* = 0.028	
LP	138.34 ± 1.75	141.55 ± 2.85	136.20 ± 3.67	*p* = 0.494; * η*^2^*p* = 0.017	
Mediodorsal paratenial reuniens	1024.70 ± 8.10	963.41 ± 13.17	1014.00 ± 16.99	*p* < 0.001; * η*^2^*p* = 0.156	C9orf72 < GeneNegative (*p* < 0.001); C9orf72 < SOD1 (*p* = 0.072)
Motor nuclei	1925.53 ± 12.99	1962.99 ± 21.13	1894.25 ± 6.39	*p* = 0.138; * η*^2^*p* = 0.047	
Sensory nuclei	869.66 ± 6.39	925.78 ± 10.40	868.47 ± 13.41	*p* < 0.001; * η*^2^*p* = 0.207	C9orf72 > GeneNegative (*p* < 0.001); C9orf72 > SOD1 (*p* = 0.004)
Intralaminar	438.53 ± 3.35	444.59 ± 5.44	438.28 ± 7.02	*p* = 0.634; * η*^2^*p* = 0.011	
Right thalamus^b^					
AV	146.04 ± 2.12	149.92 ± 3.46	140.89 ± 4.48	*p* = 0.305; * η*^2^*p* = 0.029	
LGN	195.57 ± 3.40	199.43 ± 5.56	189.18 ± 7.19	*p* = 0.552; * η*^2^*p* = 0.014	
MGN	128.02 ± 1.76	131.18 ± 2.88	123.64 ± 3.72	*p* = 0.303; * η*^2^*p* = 0.029	
Pulvinar limitans	1672.23 ± 14.36	1601.58 ± 23.48	1654.62 ± 30.35	*p* = 0.047; * η*^2^*p* = 0.072	C9orf72 < GeneNegative (*p* = 0.041)
LD	30.80 ± 0.91	32.86 ± 1.49	32.28 ± 1.92	*p* = 0.456; * η*^2^*p* = 0.019	
LP	127.60 ± 1.65	128.67 ± 2.69	133.53 ± 3.48	*p* = 0.312; * η*^2^*p* = 0.028	
Mediodorsal paratenial reuniens	1041.07 ± 7.94	1018.39 ± 12.98	1069.44 ± 16.78	*p* = 0.070; * η*^2^*p* = 0.063	
Motor nuclei	1950.55 ± 13.03	1967.71 ± 21.30	1929.43 ± 27.54	*p* = 0.565; * η*^2^*p* = 0.014	
Sensory nuclei	929.49 ± 6.36	974.89 ± 10.40	936.08 ± 13.44	*p* = 0.002; * η*^2^*p* = 0.141	C9orf72 > GeneNegative (*p* < 0.001); C9orf72 > SOD1 (*p* = 0.088);
Intralaminar	434.56 ± 3.63	451.30 ± 5.94	446.84 ± 7.68	*p* = 0.042; * η*^2^*p* = 0.075	C9orf72 > GeneNegative (*p* = 0.062);

Age = 43.43; Sex = 1.57; Education = 14.57; Left total thalamic volume = 6311.78; Right total thalamic volume = 6655.93. Post hoc univariate comparisons across groups were performed only in case of a significant multivariate omnibus test: ^a^Wilks’ Lambda = 0.584; *F* (18, 148) = 2.535; *p* = 0.001, ^b^Wilks’ Lambda = 0.590; *F* (18, 148) = 2.481; *p* = 0.001. Partial *η*^2^ effect size is interpreted as small (*η*^2^*p*  = 0.01), medium (*η*^2^*p*  = 0.06) and large (*η*^2^*p*  = 0.14)

**Fig. 2.**
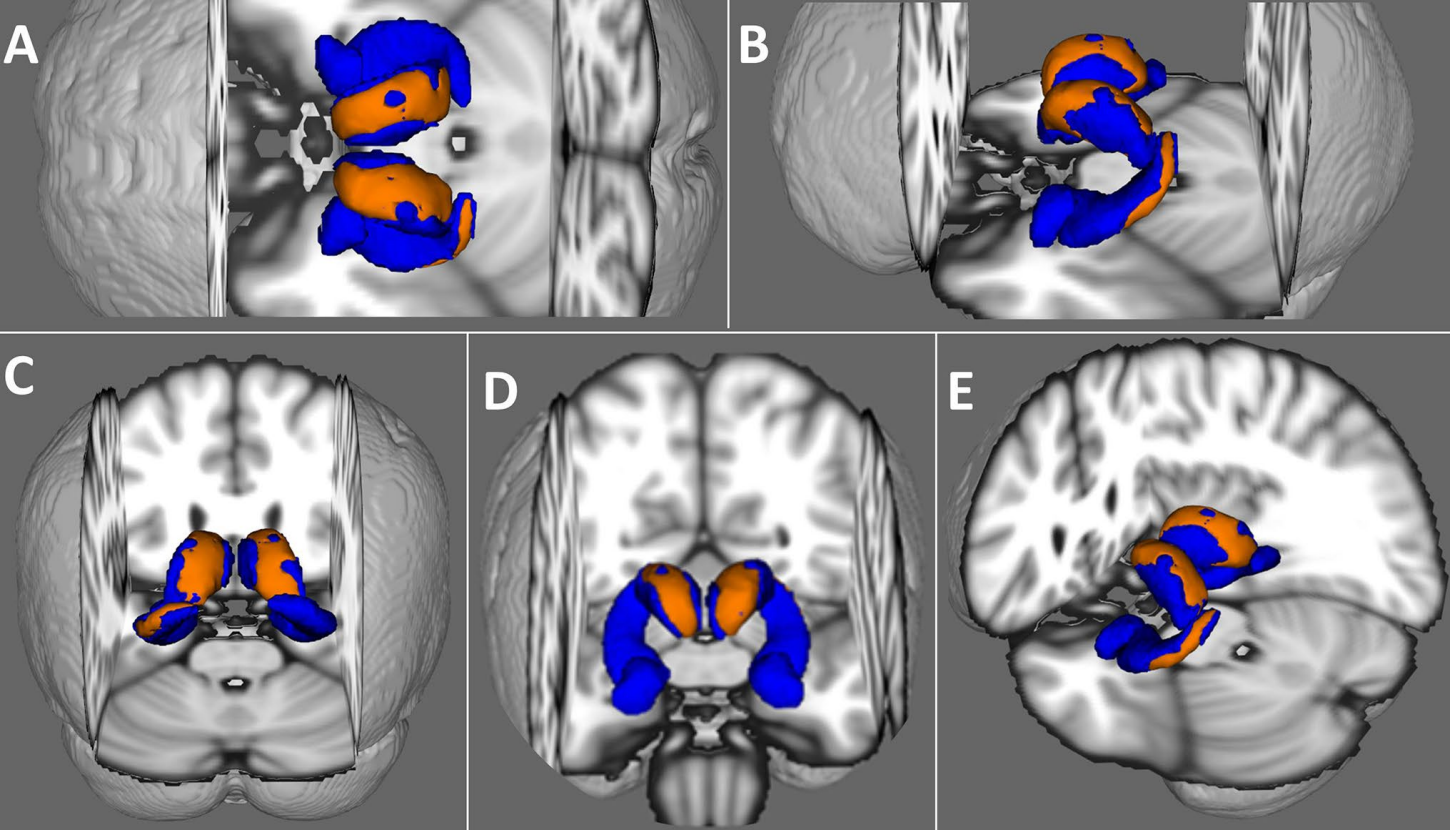
3D representation of shape deformations in C9orf72 hexanucleotide repeat expansion carriers compared to gene-negative controls at *p* < 0.05 FWE (age, sex, TIV, education corr.) in the anterior, superior, and posterior surface of both thalami as well as the lateral aspect of the left hippocampus. Blue colour represents the three-dimensional study-specific mesh of the bilateral thalami, hippocampi, and amygdalae. Orange colour represents areas of surface deformations at *p* < 0.05 FWE corr. in C9orf72 mutation carriers. *A* superior view, *B* left lateral view, *C* posterior view, *D* anterior view, and *E* left lateroposterior oblique view

**Fig. 3:**
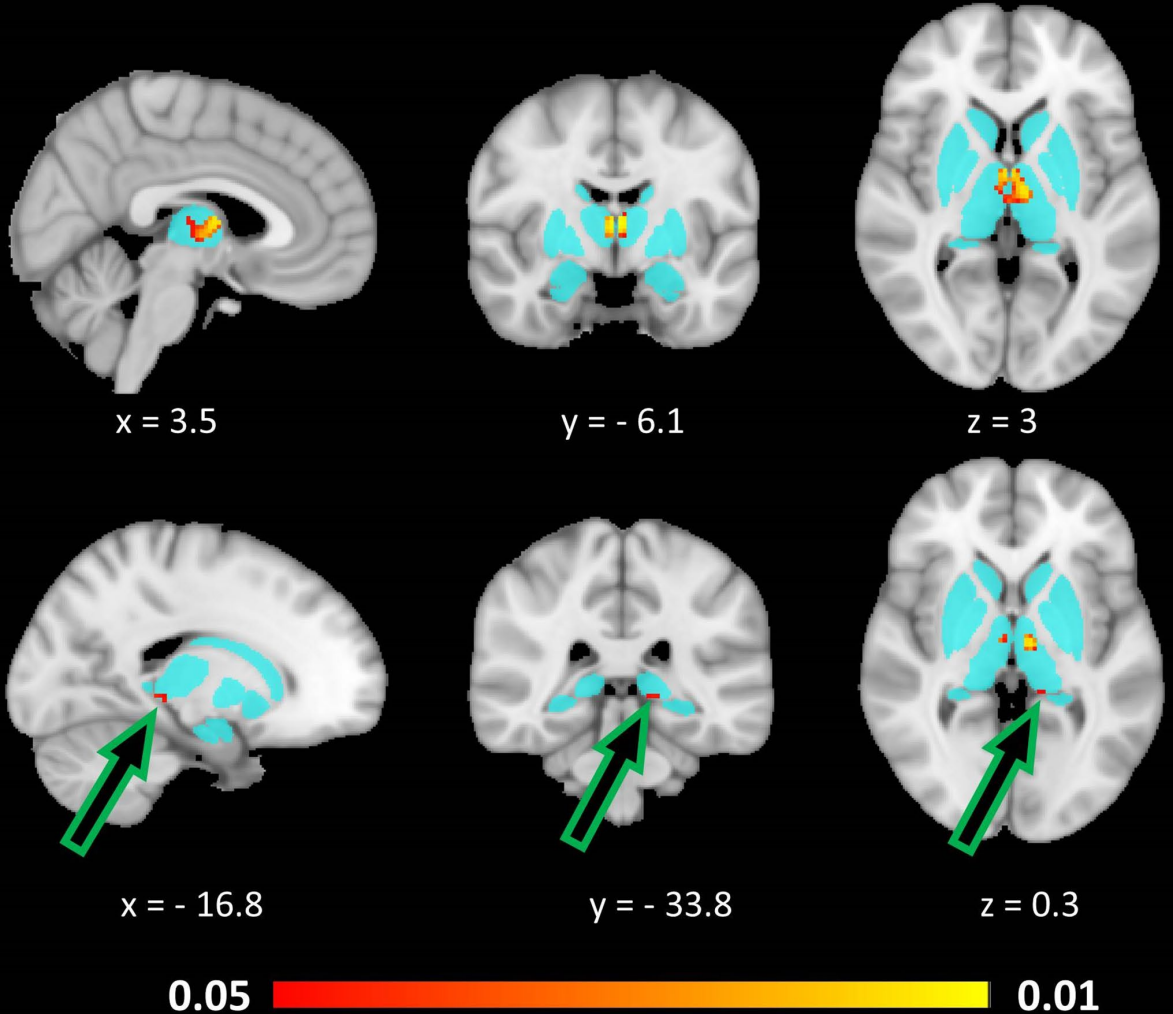
Morphometric alterations based on signal reductions in *C9orf72* hexanucleotide repeat expansion carriers compared to gene-negative controls at *p* < 0.05 TFCE corrected for age, sex, education, and TIV. The aqua blue colour underlay represents the unified basal ganglia-thalamic region-pf-interest mask and the statistically significant clusters at *p* < 0.05 TFCE are shown in red–orange. Slice coordinates are provided with reference to the Montreal Neurosciences Institute (MNI152) space. Green arrows highlight the small cluster of signal reduction in the pulvinar region of the left thalamus

Vertex analyses identified shape deformations in *C9orf72* hexanucleotide repeat expansion carriers compared to gene-negative controls in the anterior, superior, and posterior surface of both thalami as well as the lateral aspect of the left hippocampus (Fig. 2). Vertex analyses in *SOD1* carriers did not identify shape deformation compared to gene-negative controls. ROI morphometric analyses revealed bi-thalamic and left pulvinar signal reductions in *C9orf72* hexanucleotide repeat expansion carriers compared to gene-negative controls (Fig. 3). In our voxelwise analyses, the contrasts between *SOD1* carriers and gene-negative controls did not reach significance.

## Discussion

Our computational image analyses capture thalamic and hippocampal alterations in a cohort asymptomatic *C9orf72* hexanucleotide repeat expansion carriers without associated neocortical grey matter atrophy. No cortical or subcortical grey matter pathology was observed in our presymptomatic *SOD1* group.

Methodologically, our study benefits from a multiparametric approach, where data were interrogated in multiple pipelines run in different image analyses suites resulting in good anatomical concordance. The results of our post-segmentation volumetric analyses and our ROI-based morphometric subcortical analyses are relatively concordant in identifying bilateral mediodorsal thalamic atrophy (Table 3, Fig. 3.). Our results indicate that as opposed to global thalamic degeneration, selective thalamic involvement characterises the asymptomatic phase of *C9orf72*. The shape deformation identified on vertex analyses confirms focal thalamic involvement, but the nature of these analyses is that surface-projected changes are captured instead of the intra-thalamic changes described by morphometric and post-segmentation pipelines. Resting-state fMRI studies have consistently described widespread connectivity alterations in *C9orf72* mutation carriers including networks relayed through the thalamus [13, 55]. Network integrity alterations were also detected using chronnectomic approaches [56] and thalamic hypometabolism has also been consistently identified by PET studies [57, 58]. MR spectroscopy [59] identified reduced putaminal NAA/Cr, Glu/Cr and Glu/NAA ratios, and reduced thalamic Glu/NAA in asymptomatic C9orf72 mutation carriers [19]. Based on structural data, thalamic atrophy [8, 13, 16, 18, 25–28] has been previously described in presymptomatic *C9orf72* hexanucleotide carriers as well as disease burden in other subcortical grey matter structures, such as the caudate [14, 60], putamen [14], and striatum, but the predilection for specific nuclei or subregions have not been comprehensively analysed. In our study, the two main thalamic regions identified by both our volumetric and morphometric analysis streams are the mediodorsal and pulvinar regions. The physiological role of cortico-basal networks relayed though specific nuclei are fairly well established [33, 35, 61]. Thalamic atrophy has been highlighted in most genetic variants of FTD [35, 62], but medial pulvinar degeneration is thought to be relatively unique to *C9orf72* [62, 63]. The involvement of specific groups of nuclei at a presymptomatic phase is relativity novel as previous studies primarily focused on global thalamus pathology [18] or only described focal changes based on voxelwise analyses. In a particularly elegant recent study, spectral clustering, a graph-based partitioning technique was implemented which revealed posteromedial thalamic changes in asymptomatic *C9orf72* carriers. [64] The early degeneration of mediodorsal and pulvinar regions in our *C9orf72* cohort may herald future dysfunction in associated cognitive domains, such as limbic, executive, and associative processes.

It is noteworthy that the patterns of grey matter changes identified in this asymptomatic cohort, are also relatively consistent with the “post-symptomatic” signature associated of the genotype. Selective thalamic atrophy in symptomatic *C9orf72* hexanucleotide expansion carriers is evidenced by a multitude of large, prospective neuroimaging studies including both ALS and FTD phenotypes [35, 65]. Grey matter involvement in *C9orf72* carriers outside the neocortex has a predilection for the thalamus, [66–70] although hippocampal, putaminal, striatal, caudate, cerebellar, and nucleus accumbens changes are also commonly reported [14, 40, 71, 72]. Even though grey matter disease burden is thought to be more pronounced in *C9orf72*-associated ALS [71, 73], considerable subcortical grey matter pathology is also readily identified in *C9orf72*-negative ALS and PLS [40, 47, 74–77]. Basal ganglia changes have also been consistently described in sporadic patients and linked to neuropsychological and extra-pyramidal deficits [78–80]. Similar to our findings in this cohort of presymptomatic patients, a study of symptomatic *C9orf72* patients with ALS fulfilling the EL Escorial criteria [69] also identified the selective degeneration of thalamic nuclei in *C9orf72*-positive ALS preferentially involving the mediodorsal–parateniual–reuniens group of nuclei, which play a central role in executive processes. Interestingly, we have detected higher sensory nuclei volumes bilaterally in *C9orf72* hexanucleotide carriers compared to both “gene-negative” ALS kindreds and asymptomatic *SOD1* mutation carriers. While somatosensory impairment is not classically associated with the core clinical features of ALS, in symptomatic cohorts, imaging studies have consistently confirmed the involvement of somatosensory structures [34, 70]. The marked differences between *SOD1* and *C9orf72* showcase the notable heterogeneity of ALS, and given the relatively young age profile of participants, the higher volumes detected hexanucleotide repeat carriers may support the role of neurodevelopmental factors [17, 81, 82]. Our vertex analyses also highlight hippocampal atrophy in hexanucleotide carriers which is also consistent with observations from symptomatic patient groups [14, 40, 71] and in line with the reports of early memory impairment in clinical subgroups of ALS [83]. Also, these subjects exhibit nucleus reuniens involvement of a key hub of thalamic afferents to the hippocampus [84]. Thus, *C9orf72* carriers display concomitant thalamic and hippocampal alterations which are probably functionally interlinked. However, given that our hippocampal findings are unilateral, further validation is needed by larger studies.

The practical relevance of multimodal presymptomatic studies stems from the prospect of developing accurate predictive models to foretell the approximate time of phenoconversion and the likely clinical phenotype. This would enable precision care planning for individual subjects and optimised timing for clinical trial inclusion. The utility of machine learning (ML) has already been demonstrated in a variety of prognostic and diagnostic applications in ALS [85–87]. Imaging-based ML models in ALS increasingly include subcortical measures [88–91] in addition to cortical grey matter and cerebral white matter metrics [92–95]. Feature importance analyses and cluster analyses consistently confirmed the discriminatory potential of subcortical indices and integrity metrics of networks relayed through subcortical nuclei [96, 97]. In asymptomatic *C9orf72* HRE carriers, emerging spinal cord imaging techniques may be particularly useful in delineating incipient ALS from FTD [9, 98]. MRI-based predictive models have already been successfully trialled in symptomatic ALS patients [92, 99] and similar strategies could be adopted in asymptomatic cohorts to foretell likely phenoconversion.

Our results highlight the fundamental heterogeneity of genetic ALS from its earliest stages, by demonstrating the strikingly divergent disease-burden patterns between *C9orf72* and *SOD1* carriers. Despite the insights generated by a cross-sectional study, ultimately, large, multi-timepoint longitudinal studies are required to track mutation carriers from a very young age until fulfilling diagnostic criteria and beyond. The lack of longitudinal analyses is the biggest limitation of this cross-sectional study. While our descriptive statistics demonstrate focal grey matter changes, they only offer a mere snapshot in time. Characterising the evolution of grey matter alterations from birth to phenoconversion through several timepoints would reveal the full biological trajectory of these processes [100]. Importantly, the subjects of this study have not yet met relevant diagnostic criteria, and therefore, we cannot be sure whether individual hexanucleotide expansion carriers will develop a syndrome primarily consistent with ALS or FTD, although the index cases (affected, symptomatic family members) of the current study all had ALS. Finally, we acknowledge the limitations of the raw data and wish that additional spectroscopic or resting-state fMRI data would be at our disposal to comprehensively interrogate metabolic and connectomic alterations.

## Conclusions

*C9orf72* hexanucleotide repeat expansions are associated with presymptomatic thalamus and hippocampus alterations which may precede detectable neocortical involvement. The identified radiological changes may be mediated by a multitude of *C9orf72*-associated pathophysiological processes which take place decades before symptom manifestation.

## Supplementary Information

Below is the link to the electronic supplementary material.

Supplementary file1 (DOCX 30 KB)

## Data Availability

Group-level outputs, post-hoc statistics and additional information on data processing pipelines can be requested from the corresponding author. Individual-subject neuroimaging data cannot be made available due to departmental policies.

## Abbreviations

AAA: Anterior amygdaloid area
ABN: Accessory basal nucleus
ALS: Amyotrophic lateral sclerosis
AN: Attention network
ANCOVA: Analysis of covariance
AV: Anteroventral nuclei
BG: Basal ganglia
BN: Basal nucleus
C9orf72: Chromosome 9 open-reading frame 72
CA: Cornu Ammonis
CAT: Cortico-amygdaloid transition
CeM: Central medial nucleus
CL: Central lateral nucleus
CM: Centromedian nucleus
CN: Cortical nucleus
CST: Corticospinal tract
Cr: Creatine‐phosphocreatine
CT: Cortical thickness
DTI: Diffusion tensor imaging
ECAS: Edinburgh Cognitive ALS Screen
EMM: Estimated marginal mean
EPI: Echo-planar imaging
eTIV: Total intracranial volume estimates
FTD: Frontotemporal dementia
FLAIR: Fluid-attenuated inversion recovery
FOV: Field-of-view
FSL: FMRIB Software Library
FWE: Familywise error
GC-DG: Granule cell layer of dentate gyrus
GM: Grey matter
HARDI: High angular resolution diffusion imaging
HATA: Hippocampus-amygdala transition area
HC: Healthy control
HRE: Hexanucleotide repeat expansions
IR-SPGR: Inversion Recovery prepared Spoiled Gradient Recalled echo
LD: Laterodorsal nuclei
LN: Lateral nucleus
LGN: Lateral geniculate
LMN: Lower motor neuron
LP: Lateral posterior nuclei
L-SG: Limitans/suprageniculate nuclei
Lt: Left
M: Mean
MANCOVA: Multivariate analysis of covariance
MDI: Mediodorsal lateral parvocellular nuclei
MDm: Mediodorsal medial magnocellular nuclei
MGN: Medial geniculate nuclei
ML: Machine learning
MN: Medial nucleus
MND: Motor neuron disease
MNI152: Montreal Neurological Institute 152 standard space
MR: Magnetic resonance
MRS: Magnetic resonance spectroscopy
MT: Magnetisation transfer
MV-re: Reuniens/medial ventral nuclei
Myo: Myoinositol
NAA: *N*-Acetylaspartate
NODDI: Neurite orientation dispersion and density imaging
PBA: Pseudobulbar affect
Pc: Paracentral nuclei
PCL: Pathological crying and laughing
Pf: Parafascicular nuclei
PLS: Primary lateral sclerosis
PMA: Progressive muscular atrophy
PMC: Primary motor cortex
PN: Paralaminar nucleus
PPZ: Perforant pathway zone
Pt: Paratenial nuclei
pTDP-43: Phosphorylated 43 kDa TAR DNA-binding protein
PuA: Pulvinar anterior nuclei
PuI: Pulvinar inferior nucleus
PuL: Pulvinar lateral nucleus
pTDP-43: Phosphorylated 43 kDa TAR DNA- binding protein
PuM: Pulvinar medial nucleus
QSM: Quantitative susceptibility mapping
RE: Repeat expansion
ROI: Region-of-interest
Rt: Right
SBMA: Spinal and bulbar muscular atrophy / Kennedy's disease
SD: Standard deviation
SE: Standard error
SOD1: Superoxide dismutase 1
SN: Salience network
SPSS: Statistical product and service solutions
T1W: T1-weighted imaging
TE: Echo time
TFCE: Threshold-free cluster enhancement
TI: Inversion time
TIV: Total intracranial volume
TR: Repetition time
UMN: Upper motor neuron
VA: Ventral anterior nuclei
VAmc: Ventral anterior magnocellular nuclei
VBM: Voxel-based morphometry
VLa: Ventral lateral anterior nuclei
VLp: Ventral lateral posterior nuclei
VM: Ventromedial nuclei
VPL: Ventral posterolateral nuclei
WM: White matter
