# Supplementary material for: Presymptomatic grey matter alterations in ALS kindreds: a computational neuroimaging study of asymptomatic C9orf72 and SOD1 mutation carriers
Source: J Neurol. 2023 May 13;270(9):4235–47. doi: 10.1007/s00415-023-11764-5 (PMC10421803; doi:10.1007/s00415-023-11764-5)
Supplement: Supplementary file 1 — Supplementary file1 (DOCX 30 KB) [file 415_2023_11764_MOESM1_ESM.docx]

**Supplementary Table 1**: Amygdalar nuclei mm^3^ [estimated marginal means±standard error] (covariates: age, sex, education, amygdalar volume)

|  | **Gene Negative** | ***C9orf72*** | ***SOD1*** | **Statistics**  **(univariate p-value and effect size)** |
| --- | --- | --- | --- | --- |
| **Left amygdala^a^** |  |  |  |  |
| **Lateral nucleus** | 673.93±3.66 | 670.53±5.88 | 669.27±7.65 | p=0.801; η^2^p=0.005 |
| **Basal nucleus** | 443.09±1.53 | 443.30±2.45 | 441.88±3.19 | p=0.934; η^2^p=0.002 |
| **Accessory basal nucleus** | 263.65±1.72 | 264.95±2.77 | 263.39±3.60 | p=0.917; η^2^p=0.002 |
| **Anterior amygdaloid area** | 58.10±0.71 | 57.53±1.14 | 58.86±1.48 | p=0.783; η^2^p=0.006 |
| **Central nucleus** | 47.45±0.91 | 50.28±1.47 | 47.44±1.91 | p=0.262; η^2^p=0.032 |
| **Medial nucleus** | 25.37±0.74 | 28.34±1.19 | 29.03±1.54 | p=0.028; η^2^p=0.084 |
| **Cortical nucleus** | 27.32±0.49 | 28.41±0.79 | 27.38±1.03 | p=0.506; η^2^p=0.017 |
| **Corticoamygdaloid transition** | 178.40±1.23 | 173.68±1.98 | 180.77±2.58 | p=0.069; η^2^p=0.064 |
| **Paralaminar nucleus** | 50.79±0.34 | 51.08±0.55 | 50.08±0.72 | p=0.553; η^2^p=0.015 |
| **Right amygdala^b^** |  |  |  |  |
| **Lateral nucleus** | 679.17±2.91 | 675.06±4.64 | 676.94±6.15 | p=0.745; η^2^p=0.007 |
| **Basal nucleus** | 451.78±1.63 | 457.40±2.60 | 453.31±3.44 | p=0.196; η^2^p=0.039 |
| **Accessory basal nucleus** | 273.69±1.38 | 273.99±2.20 | 272.74±2.92 | p=0.943; η^2^p=0.001 |
| **Anterior amygdaloid area** | 60.90±0.71 | 61.19±1.13 | 60.99±1.50 | p=0.978; η^2^p=0.001 |
| **Central nucleus** | 51.15±0.94 | 54.06±1.50 | 50.44±1.99 | p=0.219; η^2^p=0.037 |
| **Medial nucleus** | 26.96±0.73 | 28.77±1.16 | 27.49±1.54 | p=0.426; η^2^p=0.021 |
| **Cortical nucleus** | 28.50±0.42 | 28.23±0.68 | 28.24±0.90 | p=0.928; η^2^p=0.002 |
| **Corticoamygdaloid transition** | 183.25±1.37 | 176.75±2.18 | 184.96±2.89 | p=0.029; η^2^p=0.084 |
| **Paralaminar nucleus** | 50.57±0.33 | 50.51±0.52 | 50.86±0.69 | p=0.920; η^2^p=0.002 |

*Note*. Age = 43.43; Sex(M/F) = 1.57; Education = 14.57; Left total amygdalar volume = 1768.10mm^3^; Right total amygdalar volume = 1805.96mm^3^. Post-hoc comparisons were not performed because the multivariate omnibus test was not significant: ^a^Wilks’ Lambda = 0.744; F (16, 148) = 1.477; p = 0.115, ^b^Wilks’ Lambda = 0.804; F (16, 148) = 1.068; p = 0.390. Partial η^2^ effect size is interpreted as small (η^2^p = 0.01), medium (η^2^p = 0.06) and large (η^2^p = 0.14).

**Supplementary Table 2**: Hippocampal subfield volumes mm^3^ [estimated marginal means±standard error] (covariates: age, sex, education, hippocampal volume)

|  | **Gene Negative** | ***C9orf72*** | ***SOD1*** | **Statistics**  **(univariate p-value and effect size)** |
| --- | --- | --- | --- | --- |
| **Left hippocampus^a^** |  |  |  |  |
| **Hippocampal tail** | 524.91±6.95 | 521.05±11.21 | 520.43±14.53 | p=0.935; η^2^p=0.002 |
| **Subiculum body** | 238.42±2.69 | 245.18±4.34 | 234.78±5.62 | p=0.303; η^2^p=0.029 |
| **CA1 body** | 122.69±1.97 | 116.08±3.18 | 130.16±4.12 | p=0.034; η^2^p=0.080 |
| **Subiculum head** | 198.23±2.78 | 205.22±4.49 | 199.90±5.82 | p=0.433; η^2^p=0.020 |
| **Hippocampal fissure** | 145.83±3.09 | 146.00±4.99 | 138.78±6.47 | p=0.604; η^2^p=0.012 |
| **Presubiculum head** | 147.06±1.52 | 147.40±2.44 | 144.88±3.17 | p=0.803; η^2^p=0.005 |
| **CA1 head** | 515.82±4.03 | 520.59±6.49 | 522.96±8.41 | p=0.670; η^2^p=0.010 |
| **Presubiculum body** | 163.75±3.01 | 166.93±4.85 | 153.66±6.28 | p=0.252; η^2^p=0.033 |
| **Parasubiculum** | 64.91±1.54 | 61.87±2.49 | 67.05±3.22 | p=0.430; η^2^p=0.021 |
| **Molecular layer HP head** | 335.06±1.93 | 338.08±3.12 | 333.50±4.04 | p=0.632; η^2^p=0.011 |
| **Molecular layer HP body** | 221.08±1.67 | 217.62±2.69 | 222.69±3.49 | p=0.463; η^2^p=0.019 |
| **GC ML DG head** | 151.81±1.37 | 151.76±2.21 | 153.47±2.87 | p=0.867; η^2^p=0.004 |
| **CA3 body** | 86.92±1.63 | 83.57±2.63 | 87.83±3.41 | p=0.513; η^2^p=0.016 |
| **GC ML DG body** | 131.64±1.03 | 131.66±1.66 | 130.64±2.15 | p=0.913; η^2^p=0.002 |
| **CA4 head** | 125.53±1.06 | 124.52±1.72 | 126.17±2.23 | p=0.829; η^2^p=0.005 |
| **CA4 body** | 116.39±1.07 | 115.90±1.73 | 115.91±2.24 | p=0.961; η^2^p=0.001 |
| **Fimbria** | 85.55±2.01 | 86.16±3.25 | 81.32±4.21 | p=0.629; η^2^p=0.011 |
| **CA3 head** | 119.16±1.53 | 117.74±2.47 | 119.54±3.20 | p=0.871; η^2^p=0.003 |
| **HATA** | 58.82±1.23 | 56.46±1.99 | 62.88±2.58 | p=0.166; η^2^p=0.043 |
| **Whole hippocampal body** | 1166.44±6.72 | 1163.10±10.84 | 1156.99±14.05 | p=0.825; η^2^p=0.005 |
| **Whole hippocampal head** | 1716.43±9.50 | 1723.63±15.32 | 1730.35±19.86 | p=0.790; η^2^p=0.006 |
| **Right hippocampus^b^** |  |  |  |  |
| **Hippocampal tail** | 571.38±6.87 | 563.37±11.04 | 554.73±14.47 | p=0.537; η^2^p=0.015 |
| **Subiculum body** | 247.46±2.75 | 242.84±4.41 | 240.64±5.78 | p=0.452; η^2^p=0.019 |
| **CA1 body** | 133.78±2.12 | 135.72±3.40 | 140.04±4.46 | p=0.440; η^2^p=0.020 |
| **Subiculum head** | 190.24±2.62 | 199.17±4.22 | 192.36±5.53 | p=0.212; η^2^p=0.038 |
| **Hippocampal fissure** | 153.73±3.93 | 162.28±6.32 | 145.52±8.29 | p=0.284; η^2^p=0.031 |
| **Presubiculum head** | 140.50±1.72 | 142.17±2.76 | 140.82±3.61 | p=0.878; η^2^p=0.003 |
| **CA1 head** | 533.49±3.95 | 544.07±6.35 | 547.33±8.33 | p=0.177; η^2^p=0.042 |
| **Presubiculum body** | 157.87±3.09 | 146.94±4.96 | 157.60±6.50 | p=0.178; η^2^p=0.042 |
| **Parasubiculum** | 63.46±1.52 | 58.54±2.44 | 62.72±3.19 | p=0.242; η^2^p=0.034 |
| **Molecular layer HP head** | 340.10±1.99 | 346.37±3.20 | 343.38±4.20 | p=0.245; η^2^p=0.034 |
| **Molecular layer HP body** | 234.01±1.80 | 231.76±2.90 | 235.50±3.80 | p=0.719; η^2^p=0.008 |
| **GC ML DG head** | 161.53±1.59 | 162.49±2.56 | 162.95±3.36 | p=0.902; η^2^p=0.003 |
| **CA3 body** | 98.91±1.72 | 102.18±2.76 | 98.51±3.62 | p=0.588; η^2^p=0.013 |
| **GC ML DG body** | 138.85±1.33 | 141.03±2.14 | 135.60±2.81 | p=0.335; η^2^p=0.027 |
| **CA4 head** | 133.20±1.19 | 133.86±1.92 | 134.00±2.51 | p=0.933; η^2^p=0.002 |
| **CA4 body** | 123.67±1.43 | 126.21±2.30 | 120.70±3.01 | p=0.366; η^2^p=0.025 |
| **Fimbria** | 77.26±2.25 | 71.45±3.62 | 73.09±4.74 | p=0.351; η^2^p=0.026 |
| **CA3 head** | 131.51±1.82 | 131.22±2.93 | 132.94±3.84 | p=0.935; η^2^p=0.002 |
| **HATA** | 60.51±1.16 | 58.35±1.86 | 64.84±2.44 | p=0.126; η^2^p=0.050 |
| **Whole hippocampal body** | 1211.80±7.64 | 1198.12±12.28 | 1201.67±16.10 | p=0.598; η^2^p=0.013 |
| **Whole hippocampal head** | 1754.55±9.06 | 1776.24±14.56 | 1781.33±19.08 | p=0.270; η^2^p=0.032 |

*Note*. Age = 43.43; Sex(M/F) = 1.57; Education = 14.57; Left total hippocampal volume = 3407.78mm^3^; Right total hippocampal volume = 3537.73mm^3^. Post-hoc comparisons were not performed because the multivariate omnibus test was not significant: ^a^Wilks’ Lambda = 0.608; F (36, 128) = 1.003; p = 0.476, ^b^Wilks’ Lambda = 0.707; F (36, 128) = 0.672; p = 0.916. Partial η^2^ effect size is interpreted as small (η^2^p = 0.01), medium (η^2^p = 0.06) and large (η^2^p = 0.14).
